# PATIENT PRIORITIES CARE FOR VETERANS

**DOI:** 10.1093/geroni/igad104.1421

**Published:** 2023-12-21

**Authors:** Marcia Mecca

**Affiliations:** VA Connecticut/Yale School of Medicine, West Haven, Connecticut, United States

## Abstract

People receiving care in the Veterans Affairs (VA) health system are older, have greater socioeconomic disadvantage, and more chronic conditions than those receiving healthcare outside of VA. PPC is well-suited for older adults with multiple chronic conditions and aligns with the VA’s Whole Health movement. A small VA PPC pilot study demonstrated that those receiving PPC compared to usual care had fewer medications added and more home and community services aligned with priorities. VA primary care is a cornerstone and hub for veteran care utilizing a team-based approach. A two-site randomized controlled trial exploring the implementation of PPC in VA primary care is currently recruiting veterans. The early phase of the project involved semi-structured formative assessment interviews with key stakeholders to obtain feedback and perspectives on PPC implementation in VA primary care. Interviews were audio recorded, transcribed verbatim, and analyzed using thematic analysis. Concerns elicited were time constraints, follow up and documentation of changes in priorities over time, managing patient expectation, communication between primary care and specialty care, as well as how PPC would impact quality metrics. In response to these findings, the patient priorities note template was shortened and streamlined, and the study communications workflow was adapted to align with clinician preferences. Succinct templated language for care alignment was crafted and shared with study providers. Education for clinicians highlighted ways in which priorities could be used to optimize communication. Study procedures were added to identify quality metric exclusions. The study will explore how PPC impacts veteran care and experience.

